# Bioorthogonal metabolic glycoengineering of human larynx carcinoma (HEp-2) cells targeting sialic acid

**DOI:** 10.3762/bjoc.6.24

**Published:** 2010-03-08

**Authors:** Arne Homann, Riaz-ul Qamar, Sevnur Serim, Petra Dersch, Jürgen Seibel

**Affiliations:** 1 University of Würzburg, Department of Organic Chemistry, Am Hubland, 97074 Würzburg, Germany; 2 Helmholtz-Centre for Infection Research, Inhoffenstr. 7, 38124 Braunschweig, Germany

**Keywords:** bioorthogonal metabolic glycoengineering, click chemistry, sialic acid

## Abstract

Sialic acids are located at the termini of mammalian cell-surface glycostructures, which participate in essential interaction processes including adhesion of pathogens prior to infection and immunogenicity. Here we present the synthesis and bioorthogonal metabolic incorporation of the sialic acid analogue *N*-(1-oxohex-5-ynyl)neuraminic acid (Neu5Hex) into the cell-surface glycocalyx of a human larynx carcinoma cell line (HEp-2) and its fluorescence labelling by click chemistry.

## Introduction

**Scheme 1:**
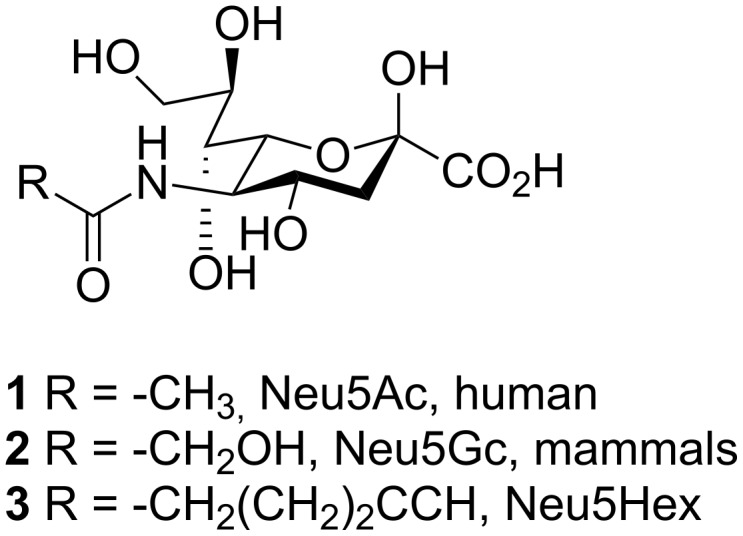
The natural forms of sialic acids, human *N*-acetylneuraminic acid (Neu5Ac, **1**) and mammalian *N*-glycolylneuraminic acid (Neu5Gc, **2**). *N*-(1-oxohex-5-ynyl)neuraminic acid (Neu5Hex, **3**) is used for bioorthogonal metabolic labelling of human larynx carcinoma (HEp-2) cells.

The surface of eukaryotic cells is heavily covered with glycan structures of various types forming the individual, dynamic glycocalyx of each cell type. These glycolipids and glycoproteins often carry sialic acids, in humans *N*-acetylneuraminic acid (Neu5Ac, **1**, Scheme 1), at their terminal position which mediate cell-cell recognition and signal transduction processes involved in infection, inflammation or tumor formation [1]. Recent studies have shown that the surface of a T-cell line (Jurkat), Chinese hamster ovary (CHO) cells, cervical adenocarcinoma (HeLa) cells as well as many other cell types can be labelled with bioorthogonal, that is metabolically inert, functionalized carbohydrates both in vitro and in vivo [2–3]. Acetylated monosaccharides, for example 2-azidoacetylamino-2-deoxy-1,3,4,6-tetraacetyl-β-D-glucopyranoside (Ac_4_GlcNAz, **16**), are believed to permeate the cell membrane by diffusion processes [4]. Recently, it was reported that neuraminic acid analogues enter the cell by pinocytosis and are incorporated into the cellular glycosylation machinery by active transporter systems [5]. In other mammals *N*-glycolylneuraminic acid (Neu5Gc, **2**, Scheme 1) corresponds to Neu5Ac **1** found in humans. Although the human gene for the synthesis of Neu5Gc **2** is inactive, small amounts of Neu5Gc **2** are also found in the human metabolism presumably dietary derived from carbohydrate salvage pathways [5–6]. The efficient uptake and incorporation of sialic acid modified in positions C-5 and C-9 into human B-lymphoma cells (BJA-B), Jurkat and others including primary cells has been demonstrated [3,7]. The sialic acid modifications influence the interaction with sialic acid binding immunoglobulin-like lectin (Siglec)-2 and infection processes of BJA-B cells by the B-lymphotrophic papovavirus [8]. It was further shown that the uptake and incorporation of alkynylated *N*-acetylmannosamine (1,3,4,6-tetraacetyl-*N*-(4-pentynoyl)mannosamine) into six different kinds of cells was more efficient than the incorporation of its azido derivative (1,3,4,6-tetraacetyl-*N*-azido-acetylmannosamine) [3]. In the current study, metabolic glycoengineering of human larynx carcinoma (HEp-2) cells with *N*-(1-oxohex-5-ynyl)neuraminic acid (Neu5Hex, **3**) is demonstrated. The bioorthogonal modification, that is the introduction of hexyne, was carried out at the sialic acid acetyl residue at position C-5 which is prone to mammalian evolution processes [5–6].

## Results and Discussion

### 

#### Synthesis of the sialic acid analogue *N*-(1-oxohex-5-ynyl)neuraminic acid (Neu5Hex, **3**)

**Scheme 2:**
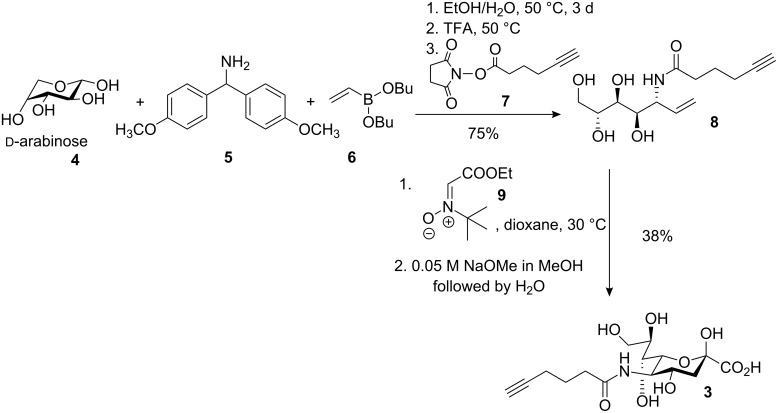
Synthesis of N-(1-oxohex-5-ynyl)neuraminic acid (Neu5Hex **3**).

The bioorthogonality of *N*-(1-oxohex-5-ynyl)neuraminic acid (**3**) was exploited to incorporate it into human larynx carcinoma (HEp-2) cells by metabolic glycoengineering. The synthesis of *N*-(1-oxohex-5-ynyl)neuraminic acid (Neu5Hex, **3**) was achieved by a previously described route [9]. The Petasis coupling was performed starting from D-arabinose (**4**), the secondary amine **5** and dibutyl vinyl boronic acid ester **6**. In situ hydrolysis of the bis(4-methoxyphenyl)methyl group with a catalytic amount of trifluoroacetic acid (TFA), followed by *N*-acylation with the activated ester **7** led to the alkyne **8** in a yield of 75% based on D-arabinose. A [3+2] cycloaddition reaction between *N*-*tert*-butyl nitrone **9** and **8** and subsequent base-catalyzed ring-opening and hydrolysis afforded *N*-(1-oxohex-5-ynyl)neuraminic acid (Neu5Hex, **3**) in 38% yield (Scheme 2).

#### Metabolic glycoengineering of human larynx carcinoma (HEp-2) cells by incorporation of *N*-(1-oxohex-5-ynyl)neuraminic acid (Neu5Hex, **3**)

**Scheme 3:**
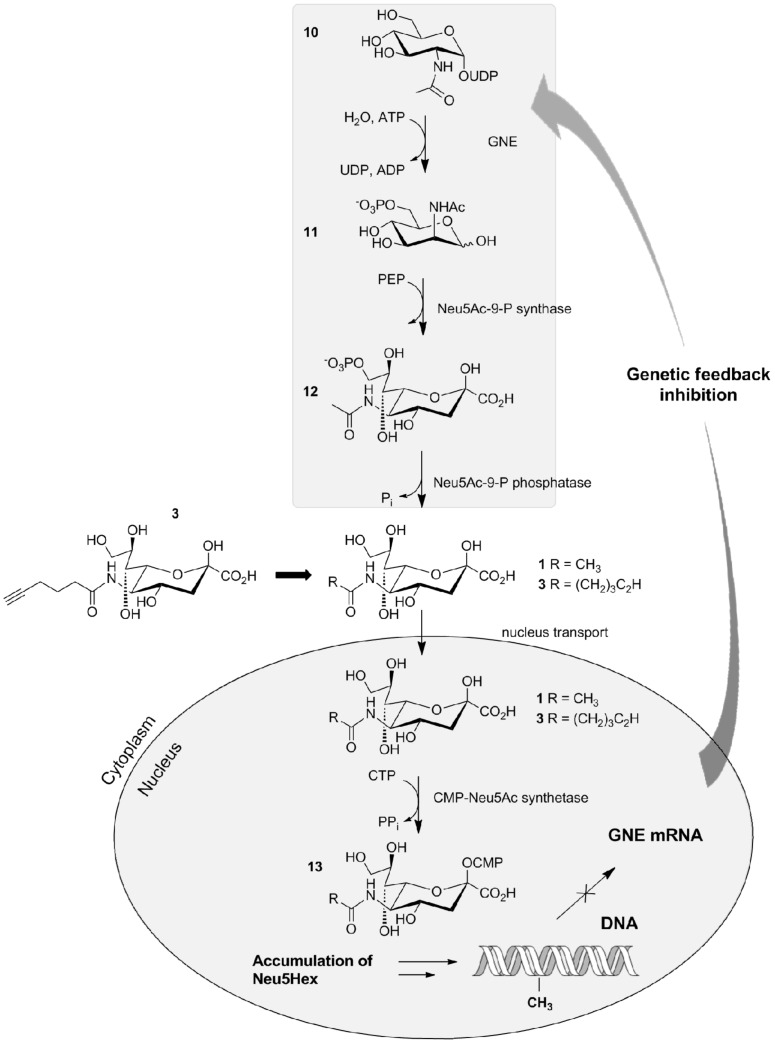
Metabolic pathway of Ac_4_GlcNAz and the genetic control of Neu5Ac **1** synthesis by feedback inhibition. The accumulation of Neu5Hex **3** is proposed by incubation of **3** with the target cell line as the synthesis of Neu5Ac **1** is down-regulated [11,13]. UDP: uridine diphosphate; GNE: UDP-*N*-acetylglucosamine 2-epimerase/*N*-acetylmannosamine kinase; ATP: adenosine triphosphate; PEP: phosphoenolpyruvate; CTP: cytidine triphosphate; PP_i_: pyrophosphate; DNA: deoxyribonucleic acid; mRNA: messenger ribonucleic acid.

The metabolic labelling of human larynx carcinoma (HEp-2) cell surfaces was carried out in order to study and characterize the influence of sialic acid in cell signalling and cell-cell interactions. HEp-2 cells were investigated because of their metabolic capability to incorporate 2-azidoacetylamino-2-deoxy-(1,3,4,6)-tetraacetyl-β-D-glucopyranoside (Ac_4_GlcNAz, **16**). The internalization of this acetylated monosaccharide was described previously as a diffusion process through the membrane of eukaryotic cells [3]. Neu5Hex (**3**) is a new substrate for metabolic glycoengineering which is proposed to be incorporated into the cell surface glycan structures. It was shown that carbohydrates in growth media contribute to alterations in glycosylation patterns in human cells [8,10]. The bifunctional enzyme UDP-*N*-acetylglucosamine 2-epimerase/*N*-acetylmannosamine kinase (GNE) is the key enzyme in sialic acid biosynthesis. The inhibitory effect of the sialic acid concentration towards the UDP-*N*-acetylglucosamine 2-epimerase/*N*-acetylmannosamine kinase (GNE) by allosteric effects is known [11]. Recently, the regulation of UDP-GlcNAc 2-epimerase/ManNAc kinase expression on the transcriptional level by DNA methylation was demonstrated [12] and a genetic feedback regulation for this process was proposed (Scheme 3) [13]. Ac_4_GlcNAz **16** or Neu5Hex **3**, respectively, were incubated with HEp-2 cells. Ac_4_GlcNAz **16** is believed to enter the cell by diffusion through the membrane, to undergo deacetylation in the cytoplasm and then incorporated into the cell surface glycoproteins and glycolipids. Alternatively, it is metabolically converted to Neu5Az [14].

**Scheme 4:**
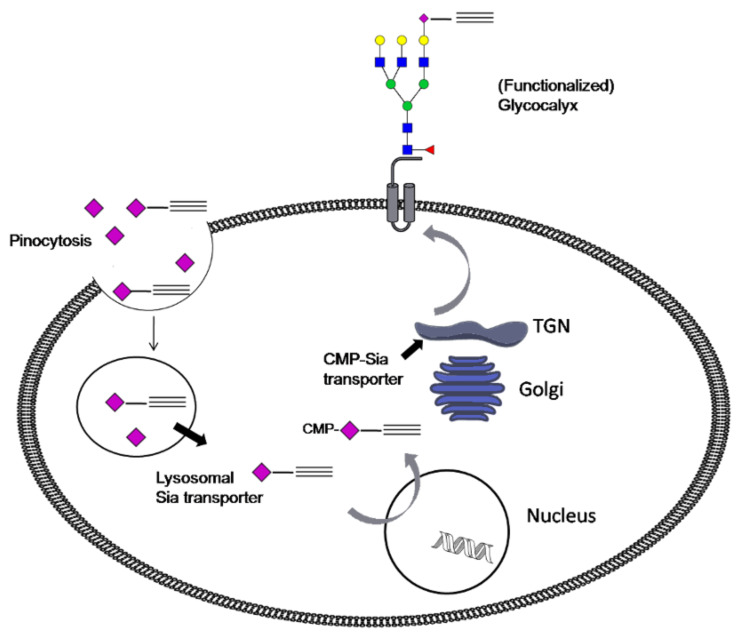
Proposed metabolic pathway of Neu5Hex **3** based on known mechanisms of Neu5Gc **2** uptake [5]. TGN: trans-Golgi network, CMP: cytidine monophosphate, Sia: sialic acid.

Neu5Hex may enter the cell by the previously described pinocytosis processes (Scheme 4) or by an, as yet, unknown internalization mechanism [5]. It is believed that Neu5Hex enters the nucleus and enhances the genetic feedback control of the GNE coding gene which blocks the synthesis of natural Neu5Ac [11,13]. Alkyne- or azide-functionalized carbohydrates in the glycocalyx are specifically addressed by complementary functionalized fluorescence agents 9-[2-carboxy-4-[(2-propyn-1-ylamino)carbonyl]phenyl]-3,6-bis(dimethylamino)xanthylium, alkynylated TAMRA or benzoic acid 2-[6-(3-azidopropanyloxy)-3-oxo-3*H*-xanthen-9-yl] 3-azidopropanyl ester, azido-fluorescein (**14**).

**Scheme 5:**
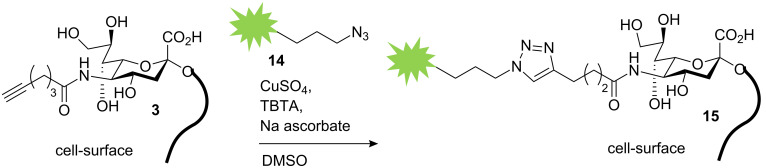
Labelling of alkynylated neuraminic acid by azido-fluorescein.

**Figure 1:**
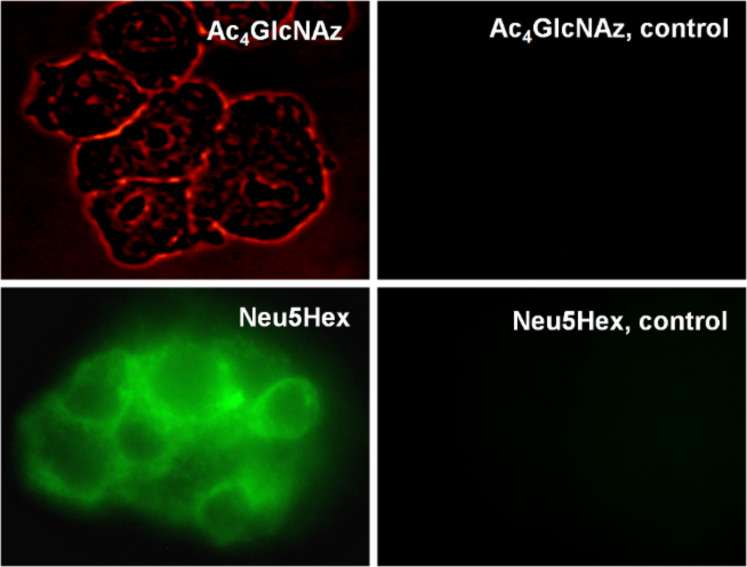
Top left: HEp-2 cells incorporated with Ac_4_GlcNAz **16,** labelled with alkynylated TAMRA at 580 nm. Bottom left: HEp-2 cells with incorporated NeuNHex **3**, labelled with azido-fluorescein **14**, at 525 nm. On the right: background and unspecific staining controls at the same wavelengths as the corresponding pictures on the left.

For the metabolic labelling of eukaryotic cells, HEp-2 cells were incubated in Dulbecco's modified Eagle's medium (DMEM) with 10% fetal calf serum (FCS). At 80% confluence they were split into 6-well plates with DMEM containing the functionalized carbohydrates (Ac_4_GlcNAz **16** or Neu5Hex **3**, 25 µM, 48 h). HEp-2 cells were harvested with a cell scraper, not trypsin, in order to preserve the partially protein-coupled glycocalyx. To highlight the successful incorporation of the azide and alkyne functionalities into the glycocalyx of HEp-2 cells, the fluorescence labelling reaction was performed according to a modified protocol of the [3+2] triazole cycloaddition [15–16]. The appropriate functionalized fluorescent detection molecule and the conditions for the click reaction (CuSO_4_, sodium ascorbate and tris[(1-benzyl-1*H*-1,2,3-triazol-4-yl)methyl]amine, TBTA) were applied in dimethylsulfoxide (DMSO) (Scheme 5). After one hour, the cells were analyzed by microscopy (phase contrast) at the appropriate wavelength for fluorescence imaging. Although the incubation of HEp-2 in DMSO and in the presence of copper ions is cytotoxic, the fluorescence in the labelled glycocalyx was clearly detectable. In order to analyze the natural background fluorescence of HEp-2, one sample was incubated without any additional carbohydrates. The cells were analyzed by fluorescence microscopy (580 nm for TAMRA staining and at 525 nm for fluorescein). At either wavelength, the negative control does not show any significant background fluorescence (Figure 1). In both the Neu5Hex fed HEp-2 and the incubation with Ac_4_GlcNAz a clear staining of the cellular glycocalyx at the expected wavelengths was observed.

## Conclusion

Sialic acids are prominent sugars which are located in the terminal position on cell-surface glycans. Although it has been known for many years that sialic acids are involved in myriads of interaction processes including viral infections such as the emerging flu variants, their biological role on cell surfaces of different cell lines and at different development states remains unclear. As new techniques for probing glycans have evolved only relatively recently, more information about the fundamental biological functions of carbohydrate structures can be obtained. Therefore we introduced metabolic glycoengineering of the human larynx carcinoma cell line HEp-2. The incorporation and cell surface presentation of Ac_4_GlcNAc **16** as well as the new substrate Neu5Hex **3** was successful. The copper-catalyzed [3+2] triazole formation (“click reaction”) proved very useful for the cell surface labelling because of its bioorthogonality. The incubation of HEp-2 cells with the sialic acid analogue Neu5Hex **3** guarantees its direct incorporation into the cell surface glycan patterns bypassing metabolic bottlenecks. Furthermore, the described genetic feedback inhibition by sialic acid leading to an accumulation of the fed Neu5Hex **3** ensures an efficient integration into the cell surface glycocalyx. A drawback of the reaction parameters and compounds used for the click reaction is the cytotoxicity of DMSO and copper. But this problem for in vivo labelling can be overcome by different reaction conditions and different detection molecules. For example, the strain-promoted click reaction with difluorinated cyclooctyne (DIFO) and cell-surface azido-glycans introduced recently has been proven to be suitable for in vivo labelling [14,17–18].

## Experimental

2-azidoacetylamino-2-deoxy-1,3,4,6-tetraacetyl-β-D-glucopyranoside (**16**) was synthesized as described previously [9]

### 

#### *N*-(1*R*,2*R*,3*S*,4*R)-*Hex-5-yonic acid (2,3,4,5-tetrahydroxy-1-vinyl-pentyl)-amide (**8**)

A solution of D-arabinose (1.09 g, 5.73 mmol, **4**), 4,4’-dimethoxybenzhydrylamine (1.39 g, 5.73 mmol, **5**) and vinyl boronic acid dibutyl ester (2.51 g, 11.46 mmol, **6**) in aqueous ethanol (60 mL, ethanol/H_2_O = 4:1) was stirred at 50 °C for 72 h. TFA (1.79 ml) was added and the reaction mixture stirred for a further 16 h. The solvent was evaporated and the residue dissolved in MeOH (30 mL). Sodium bicarbonate (974 mg, 11.59 mmol) and 5-hexynoic acid, 2,5-dioxo-1-pyrrolidinyl ester [15] (950 mg, 8.5 mmol) was added and the solution stirred for 1 h at room temperature. The solids were removed by filtration, the filtrate was dried and the residue purified by flash chromatography on silica gel (eluted with CH_2_Cl_2_ and MeOH) to afford **8** as white solid in 75% yield. *R*_f_ = 0.34 (CH_2_Cl_2_/MeOH, 7:1); [α]_D_^20^ : +19.8 (*c* 1, MeOH); ^1^H NMR (400 MHz, CD_3_OD) δ = 6.05 (ddd, *J* = 17.29, 10.53, 5.66 Hz, 1H, 1”-H), 5.27 (td, *J* = 17.29, 1.52 Hz, 1H, 2”-H), 5.22 (td, *J* = 10.54, 1.52 Hz, 1H, 2”-H), 4.56 (m, 1H, 1-H), 3.84–3.50 (m, 5H, 5-H_2_, 4-H_2_, 3-H, 2-H), 2.50 (t, *J* = 7.26 Hz, 2H, 2’-H_2_), 2.30–2.20 (m, 3H, 6’-H, 4’-H_2_), 1.90–1.70 (m, 2H, 3’-H_2_); ^13^C NMR: (100 MHz, CD_3_OD) δ = 175.29 (CO), 137.04 (C-1”), 116.65 (C-2”), 84.12 (C-5’), 72.46, 72.11, 71.55 (C-4, C-3, C-2), 70.34 (C-6’), 64.94 (C-5), 55.11 (C-1), 35.89 (C-2’), 25.77 (C-3’), 18.58 (C-4’); MS (ESI): *m/z* [M+Na]^+^ calculated for C_13_H_20_NO_5_[Na]^+^, 294.14, found 294.1.

#### Synthesis of *N*-(hex-5’-ynoyl)neuraminic acid (1”*S*,2”*R*,3”*S*,4”*R*)-2-*tert*-butyl-5-(1”-(hex-5’-ynoyl)amino-2’’,3’’,4’’,5’’-tetrahydroxy-pentyl)-isoxazolidine-3-carboxylic acid ethyl ester

Polyhydroxy olefin (1.50 g, 5.53 mmol, **8**) and nitrone (2.01 g, 11.6 mmol, **9**) in dioxane (100 mL) were stirred at 30 °C for 14 d. After complete conversion of the starting material as monitored by TLC, the solvent was removed at reduced pressure. The residue was purified by normal silica gel chromatography (MeOH/CH_2_Cl_2_, 1:10 to 1:5) to afford the ester as colourless oil (2.06 g, 4.51 mmol) in 82% yield. *R*_f_ = 0.42 (CH_2_Cl_2_/MeOH, 7:1); [α]_D_^20^ = +7.2 (*c* 1, MeOH); ^1^H NMR (400 MHz, CD_3_OD) δ = 4.66–4.61 (dt, *J =* 8.44, 1.56 Hz, 1H, 5-H), 4.14–4.08 (dq, *J* = 7.16, 1.54 Hz, 2H, OC*H_2_*CH_3_), 3.91–3.87 (t, *J* = 8.52 Hz, 1H, 1’’-H), 3.86–3.47 (m, 5H, 5’’-H_2_, 4’’-H, 3’’-H, 2”-H), 3.32–3.28 (dd, *J* = 8.70, 0.83 Hz, 1H, 3-H), 3.23–3.20 (m, 1H, 6’-H), 2.65–2.57 (m, 1H, 4-H_a_), 2.40–2.34 (t, *J* = 7.18 Hz, 2H, 4’-H_2_), 2.18–2.06 (m, 3H, 4-H_b_, 2’-H_2_), 1.79–1.71 (m, 2H, 3’-H_2_), 1.20–1.15 (t, *J* = 7.12 Hz, 3H, OCH_2_*CH_3_*), 1.05 (s, 9H, C(CH_3_)_3_); ^13^C NMR (75 MHz, CD_3_OD) δ = 177.07 (CONH), 174.22 (COO), 84.19 (C-5’), 77.34 (C-5), 72.15, 71.42, 70.63 (C-4’’, C-3’’, C-2”), 70.35 (C-6’), 65.27 (C-5’’), 62.50 (OCH_2_CH_3_), 62.23 (C-3), 61.21 (*C*(CH_3_)_3_, 53.71 (C-1’’), 39.07 (C-4), 35.92 (C-2’), 25.91 (C-4’), 25.91 (C(*C*H_3_)_3_), 25.85 (C-3’), 14.45 (OCH_2_*C*H_3_); MS (ESI): *m/z* [M+Na]^+^ calculated for C_21_H_36_N_2_O_8_[Na]^+^ 467.2, found 467.2.

#### *N*-(Hex-5’-ynoyl)neuraminic acid (**3**)

Isoxazoline (2.50 g, 5.63 mmol) and NaOMe (0.74 mL of 5.4 M solution in MeOH) in anhydrous MeOH (100 mL) were stirred at room temperature overnight. Water (100 mL) was added and the solution stirred for further 24 h. The mixture was then neutralized with acidic ion exchange resin containing formate ions (Amberlyte). The solvent was removed under reduced pressure and the crude product subjected for size exclusion chromatography with Biogel P2 (Bio-Rad) to afford pure **3** (934 mg) in 46% yield. *R*_f_ = 0.29 (CH_2_Cl_2_/MeOH, 5:2); [α]_D_^20^ = -19.04 (*c* 1, H_2_O); ^1^H NMR (300 MHz, CD_3_OD), β-anomer: δ = ppm 4.09–4.02 (m, 1H, 4-H), 4.03–4.00 (d, *J* = 10.74 Hz, 1H, 6-H), 3.87–3.81 (t, *J* = 10.29 Hz, 1H, H-5), 3.81–3.79 (dd, *J* = 11.47, 2.74 Hz, 1H, 9-H_a_), 3.74–3.89 (m, 1H, 8-H), 3.64–3.60 (dd, *J* = 11.21, 5.60, 1H, 9-H_b_), 3.52–3.49 (d, *J* = 9.35, 1H, 7-H), 3.23–3.20 (m, 1H, 6’-H), 2.42–2.38 (t, *J* = 7.35 Hz, 2H, 4’-H_2_), 2.26–2.20 (m, 4H, 4-H_a_, 4-H_b_, 2’-H_2_), 2.17–2.11 (dd, *J* = 12.83, 4.87, H-3eq), 1.86–1.80 (m, 3H H-3ax, 3’-H_2_); ^13^C NMR (75 MHz, CD_3_OD), δ = 177.00 (2 × CONH), 173.49 (COOH), 96.49 (C-1), 84.05 (C-5’), 72.03 (C-8), 71.55 (C-6), 70.08 (C-7), 70.03 (C-6’), 67.63 (C-4), 64.68 (C-9), 53.94 (C-5), 40.94 (C-3), 35.67 (C-2’), 25.64 (C-3’), 18.49 (C-4’); MS (ESI): *m/z* [M-H]^-^ calculated for C_14_H_21_NO_9_[H]^-^ 360.13, found 360.2.

#### Benzoic acid 2-[6-(3-azidopropanyloxy)-3-oxo-3*H*-xanthen-9-yl] 3-azidopropanyl ester, azido-fluorescein (**14**)

Iodopropyl azide (210 mg, 26 mmol) was added to a solution of fluorescein (1g, 2.6mmol) in a mixture of distilled THF/MeOH (1:1, 25 mL) and the reaction mixture stirred overnight. The crude mixture was concentrated under reduced pressure, diluted with water and extracted with EtOAc (3 × 25 mL). The combined organic layers were dried, concentrated followed and purified by flash chromatography to afford the pure required product (1.1 g, 2.2 mmol) in 84% yield. *R*_f_ = 0.34 (EtOAc), ^1^H NMR (400 MHz, CDCl_3_), δ = 8.18 (dd, *J =* 7.80, 1.38 Hz, 1H), 7.65 (m, 2H, 4-H, 5-H), 7.25 (dd, *J =* 7.55, 1.23 Hz, 1H, 3-H), 6.90 (d, *J =* 2.44 Hz, 1H, 5’’’-H), 6.82 (d, *J =* 8.91 Hz, 1H, 8’’’-H), 6.78 (d, *J =* 9.71 Hz, 1H, 1’’’-H), 6.68 (dd, *J =* 8.91, 2.44 Hz, 1H, 7’’’-H), 6.47 (dd, *J =* 9.71, 1.97 Hz, 1H, 2’’’-H), 6.38 (d, *J =* 1.97 Hz, 1H, 4’’’-H), 4.10 (t, *J =* 5.95 Hz, 2H, OC*H*_2_), 4.01 (m, 2H, OC*H*_2_), 3.47 (t, *J =* 6.50 Hz, 2H, C*H*_2_N_3_), 2.99 (m, 2H, C*H*_2_N_3_), 2.03, 1.55 (2m, 4H, 2’’-H_2_, 2’’’-H_2_). ^13^C NMR (101 MHz, CDCl_3_), δ = 185.56 (C-3’’’), 165.23 (C-1’), 163.10 (C-6’’’), 158.70 (C-4a’’’), 154.12 (C-5a’’’), 149.67 (C-9a’’’), 134.15 (C-2), 132.78, 131.31, 130.49, 130.11, 130.08, 129.72, 128.90 (C-3, C-4, C-5, C-6, C-1’’’, C-7’’’, C-8’’’), 130.27 (C-9’’’), 117.71, 114.91 (C-1, C-8a’’’), 113.55 (C-5’’’), 105.88 (C-2’’’), 100.93 (C-4’’’), 65.38 (C-1’’’’), 62.37 (C-1’’), 47.92 (C-3’’’’), 47.77 (C-3’’), 28.45 (C-2’’’’), 27.76 (C-2’’).

#### Cultivation and metabolic labelling of HEp-2 cells

Human larynx carcinoma (HEp-2) cells were cultivated in Dulbecco's modified Eagle's medium (DMEM) containing 10% fetal calf serum (FCS) at 37 °C under a 5% CO_2_ atmosphere. At 80% confluence the medium was discarded and the cells washed with PBS buffer (Gibco). After the addition of 1.5 ml of a trypsin/EDTA mixture, the cells were detached for 5 min at 37 °C. They were supplied with 8.5 ml of fresh medium and split in a ratio of 1:10.

For the metabolic labelling, HEp-2 cells were cultivated as described above. Subsequently, at 80% confluence they were seeded into 6-well dishes and incubated in 2 ml of the medium described above. The medium contained 25 μM of the modified carbohydrate to be incorporated (Ac_4_GlcNAz **16** or Neu5Hex **3**). The incubation time was 48 hours. The cells were detached using a cell scraper in order to retain the glycocalyx. 150 μL from each well was transferred into an 8-well microscopy cultivation slide and filled with 150 μL of the fresh medium. The cells were cultivated at the described growth conditions until reattachment. The medium was discarded and the cells were washed several times with PBS buffer (Gibco). The labelling reaction was performed in the dark with 2 mM of the complementary labelling molecule 9-[2-carboxy-4-[(2-propyn-1-ylamino)carbonyl]phenyl]-3,6-bis(dimethylamino)xanthylium, alkynylated TAMRA or azido-fluorescein **14**) with 2 mM CuSO_4_, 10 mM sodium ascorbate and 2 mM Tris-[(1-benzyl-1*H*-1,2,3-triazol-4-yl) methyl]amine (TBTA) in DMSO. After 1 h each well was washed several times with DMSO/water (1:1) and subsequently examined by fluorescence microscopy.
